# Repairing Qinling Giant Panda Skin Wounds Using Adipose Mesenchymal Stem Cell-Derived Extracellular Vesicles

**DOI:** 10.3390/ani15091270

**Published:** 2025-04-29

**Authors:** Suhua Gong, Hongyu Niu, Yanni Jia, Mengjie Liu, Xiaoyu Ren, Danhui Zhang, Jiena Shen, Chuangxue Yang, Yinghu Lei, Pengpeng Zhao, Pengfei Lin

**Affiliations:** 1 College of Veterinary Medicine, Northwest A&F University, Yangling 712100, China; gongsuhua@nwafu.edu.cn (S.G.); niuhongyu@nwafu.edu.cn (H.N.); ynj@nwafu.edu.cn (Y.J.); liumengjie0823@163.com (M.L.); 13772058591@163.com (X.R.); 2 Research Center for the Qinling Giant Panda, Rescue Base of Rare Wild Animals in Shaanxi Province, Louguantai, Xi’an 710402, China; zhangdanhui2006@126.com (D.Z.); 15902930258@163.com (J.S.); 13509185620@163.com (Y.L.); 3 Louguantai State Owned Ecological Experimental Forest Farm in Shaanxi Province, Zhouzhi, Xi’an 710402, China; 18729016980@163.com

**Keywords:** Qinling giant panda, adipose mesenchymal stem cells, extracellular vesicles, proteomics, skin wound repair

## Abstract

The Qinling giant panda has a high susceptibility to skin damage, which affects its survival. Although their healing efficacy in panda injuries remains unexplored, extracellular vesicles from adipose-derived mesenchymal stem cells (ADMSC-EVs) have shown promise in regenerative medicine. In this study, ADMSC-EVs were successfully obtained from Qinling giant pandas using ultracentrifugation, and proteomic techniques were used to analyze their composition and function. Primary skin fibroblasts from Qinling giant pandas were isolated and cultured to explore the effects of ADMSC-EVs on cell proliferation and migration. Additionally, a mouse model of skin injury was used to assess their wound healing effects. The ADMSC-EVs contained various substances, particularly proteins, with fifty unique proteins involved in transport, catabolism, and signal transduction identified. The application of ADMSC-EVs in a mouse model accelerated wound healing and promoted the regeneration of the epidermal and dermal layers. It facilitated the repair of skin appendages, including hair follicles and sebaceous glands. Additionally, ADMSC-EVs enhanced collagen deposition, stimulated angiogenesis, and reduced inflammation. Our findings confirm that ADMSC-EVs significantly improve skin healing, thus supporting the theoretical framework for the clinical use of giant panda extracellular vesicles and underscoring their potential for preserving the genetic resources of the Qinling giant panda.

## Simple Summary

The Qinling giant panda is a rare species that is vulnerable to skin injuries, which can threaten its health and survival. This study focuses on finding a way to help these pandas recover from skin damage. Researchers have explored the use of exosomes, tiny particles released from fat-derived stem cells, which have shown potential in promoting healing. By extracting these exosomes from the fat cells of Qinling giant pandas, the team tested their effects on skin cells and in a mouse model of skin injury. The results show that these exosomes help speed up the healing process, reduce inflammation, and improve skin repair. This research opens up new possibilities for treating skin injuries in pandas and may contribute to protecting the species and its genetic resources.

## 1. Introduction

The giant panda (*Ailuropoda melanoleuca*) has an evolutionary history spanning 7–8 million years, rendering it one of the most ancient species and symbolizing the global urgency of biodiversity conservation [1]. Found exclusively in Shaanxi Province (China), the Qinling subspecies of the giant panda is particularly endangered and distinctive from other populations. Thus, increased conservation efforts are imperative to protect this unique variant of the giant panda [2].

The daily activities of the giant panda often result in varying degrees of skin damage, particularly on its back, and lesions that compromise the integrity of the skin barrier. This sensitivity to external influences significantly increases the risk of infection, posing a serious threat to the health and survival of both wild and captive giant pandas [3]. Conventional treatments for wildlife injuries are currently limited by low healing rates, post-injury functional loss, irritations from unstable chemical properties, and extensive scarring [4,5,6]. Therefore, exploring innovative treatments for giant panda injuries is crucial for improving healing outcomes, reducing disturbances and stress, and conserving this species.

Mesenchymal stem cells (MSCs) are highly versatile adult stem cells found abundantly in various tissues, including bone marrow, umbilical cord blood, dental pulp, and adipose tissue [7]. Owing to their genetic stability, multilineage differentiation potential, and low immunogenicity, MSCs are extensively utilized in the treatment of injuries and degenerative diseases. With increasing potential in the medical and veterinary fields, they are often used in immune regulation and cancer therapy [8]. In 2013, researchers successfully isolated bone marrow mesenchymal stem cells (BM-MSCs) from the bone marrow of two stillborn giant pandas [9]. Wang et al. elucidated that basic fibroblast growth factor (bFGF) significantly enhances the proliferation of giant panda BM-MSCs through the activation of the extracellular signal-regulated kinase (ERK) signaling pathway [10]. Liu et al. subsequently reported the isolation of umbilical cord mesenchymal stem cells (UC-MSCs) from neonatal giant pandas, demonstrating differentiation potentials comparable to those of mesenchymal stem cells from other species. Additionally, both bFGF and epidermal growth factor (EGF) were identified as effective proliferative factors for UC-MSCs [11]. MSCs derived from diverse tissues represent crucial cellular models for the conservation of giant panda genetic resources, as well as for the investigation and treatment of diseases affecting this endangered species. Recently, extracellular vesicles (EVs) secreted by MSCs have attracted attention in the field of regenerative medicine. They are tiny extracellular sacs with diameters ranging from 30 to 200 nm that can encapsulate and transport complex biomolecules, including proteins, RNA, and DNA [12,13]. EVs enhance intercellular communication and bolster regenerative processes, thereby mimicking the therapeutic effects of MSCs [14]. EV therapy offers enhanced safety (reducing immune rejection and tumorigenesis risk) and the potential for targeting specific cells or damaged tissues for therapeutic purposes, compared to traditional stem cell treatment, while carrying various bioactive molecules that promote cell repair and immune modulation [15,16]. Utilizing MSC-derived EVs represents a pioneering therapeutic approach with potential benefits in minimizing the risks and complications associated with cell-based therapies [17].

Adipose tissue has emerged as a particularly advantageous source of MSCs, owing to its accessibility. Certain studies have indicated that the concentration of adipose-derived MSCs (ADMSCs) is approximately 500 times higher than that of bone marrow [18,19]. EVs derived from ADMSCs (ADMSC-EVs) can effectively reduce inflammation at injury sites [20]. For example, wounds treated with ADMSC-EVs have exhibited a significant reduction in inflammatory cell infiltration within the healing tissues of health individuals [21]. In pathological conditions such as diabetes, ADMSC-EV treatment significantly lowered the serum levels of IL-6, IL-1β, and TNF-α in volunteers, demonstrating its potential for clinically managing inflammation and enhancing tissue repair [22]. Recently, ADMSCs and their EVs have attracted attention in the veterinary field because of their immunomodulatory, anti-inflammatory, and tissue-regenerative capabilities [23]. They have been applied in the treatment of equine tendon injuries, chronic gingivostomatitis in cats, inflammatory bowel disease, and skin disorders in both canine and feline patients [24,25,26]. However, studies on the extraction and application of ADMSC-EVs in giant pandas are limited, and their effectiveness in treating giant panda injuries remains unknown.

In this study, EVs were extracted and characterized from the ADMSCs of Qinling giant pandas. Proteomic technologies were employed to analyze their protein composition and compare them with those derived from human MSCs (hMSCs). The effects of ADMSC-EVs on the proliferation and migration of giant panda fibroblasts were investigated, and their therapeutic efficacy was assessed in a murine model of skin injury. This study aimed to explore the potential value of ADMSC-EVs in healing Qinling giant panda injuries.

## 2. Materials and Methods

### 2.1. Ethics Statement

All experimental protocols received approval from the Animal Management and Ethics Review Committee of Northwest A&F University and the Qinling Giant Panda Research Center in Shaanxi. All procedures were performed in accordance with the Laboratory Animal Management Measures of Northwest A&F University and the Guidelines for the Care and Use of Laboratory Animals (ISBN-10: 0-309-15396-4), and methods are reported in compliance with the ARRIVE guidelines (https://arriveguidelines.org (accessed on 23 August 2024)) to ensure clarity and reproducibility.

### 2.2. Cell Isolation and Culture

The ADMSCs and dermal fibroblasts used in this study were isolated from a stillborn giant panda fetus at 106 d of gestation, obtained from the Shaanxi Qinling Giant Panda Research Center. The fetus was washed three times with sterile physiological saline, immersed in 75% ethanol for 90 s, and then washed three times with PBS containing penicillin (100 U/mL)–streptomycin (100 μg/mL).

#### 2.2.1. Isolation of ADMSCs

Under sterile conditions, subcutaneous adipose tissue was dissected from the stillborn fetus and cut into 1 mm^3^ pieces. The tissue was placed in a 15 mL sterile centrifuge tube, and 2 mL of type IV collagenase (5 mg/mL) was added for digestion at 37 °C for 45 min, with gentle agitation every 5 min. Once a viscous suspension formed, 8 mL of DMEM/F12 medium containing 10% fetal bovine serum (FBS) (Gibco, Grand Island, NY, USA, #A2720-801) was added to halt digestion. The suspension was filtered through a 70 μm cell strainer to remove undigested tissue and centrifuged at 1000× *g* for 10 min, and the cell pellet was resuspended and seeded into a 6-well culture plate at 5 × 10^4^ cells per well. After 48 h, the medium was replaced, removing non-adherent cells. Subsequent medium changes were performed every 24 h.

The ADMSCs were confirmed by RT-PCR for the expression of *CD73*, *CD90*, and *GNL3* markers. The primary ADMSCs were cultured for 48 h, and total RNA was extracted using Trizol. The first-strand cDNA was synthesized using a reverse transcription kit (Accurate Biology, Changsha, China; #AG11615). PCR primers are listed in Supplementary Table S1. PCR was performed using PrimeSTAR GXL Premix DNA Polymerase (Takara Bio, Dalian, China; #R051A) according to the manufacturer’s protocol, to assess the expression of the target fragments.

#### 2.2.2. Isolation of Dermal Fibroblasts

Dermal tissue was carefully dissected from the stillborn fetus, washed three times with PBS containing penicillin–streptomycin, and cut into 1 mm^3^ pieces, which were placed into a 60 mm culture dish. Next, 3 mL of DMEM containing 10% FBS was added, ensuring that the tissue remained submerged. Cell migration was monitored daily, and the medium was replaced every 48 h.

All cells were cultured at 37 °C in a 5% CO_2_ humidified incubator.

### 2.3. Isolation of EVs Derived from the ADMSCs of Qinling Giant Panda

EVs were isolated from the ADMSCs at passages 3–7 using an ultracentrifugation-based protocol, as previously described [27]. When the cells reached approximately 80% confluence, the conditioned medium was collected and subjected to sequential centrifugation steps to remove cells, debris, and larger particles. Initially, the medium was centrifuged at 500× *g* for 15 min at 4 °C, followed by the careful transfer of the supernatant to a new tube and centrifugation at 2000× *g* for 15 min at 4 °C to further eliminate cellular debris. The resulting supernatant was then centrifuged at 10,000× *g* for 30 min at 4 °C. Subsequently, the supernatant was collected and subjected to ultracentrifugation at 100,000× *g* for 70 min at 4 °C (Optima XPN-100 ultracentrifuge, Beckman Coulter, Brea, CA, USA). The supernatant was carefully discarded, and the EV pellet was resuspended in 200 µL of Dulbecco’s phosphate-buffered saline (D-PBS) (Solarbio, Beijing, China, #D1040). A small volume of the obtained exosome sample was mixed with twice its volume of RIPA lysis buffer, vortexed, and then centrifuged at 12,000× *g* for 15 min at 4 °C. The protein concentration was subsequently determined using the BCA assay, and the exosome quantity was assessed based on the protein concentration.

### 2.4. Characterization of EV

#### 2.4.1. Transmission Electron Microscopy (TEM) Analysis of EVs

A 10 µL EV sample was pipetted onto a piece of Parafilm. A copper grid was carefully placed face-down onto the droplet and incubated for 3 min to allow EV adsorption. The excess liquid was gently removed using filter paper. The grid was then stained with 2% uranyl acetate for 45 s, followed by careful removal of excess stain with filter paper. Finally, the grid was air-dried at room temperature. The morphology of the EVs was examined using a transmission electron microscope (Tecnai G2 Spirit BioTWIN, FEI, Hillsboro, OR, USA).

#### 2.4.2. Analysis of EV Particle Size Distribution

The particle size distribution of the EVs was analyzed using the ZEN3600 particle size analyzer (Malvern Instruments, Worcestershire, UK). Next, 20 µL of the extracted EV sample was taken and diluted with PBS to a final volume of 1 mL for analysis. The diluted samples were analyzed at room temperature. To ensure consistency, three repeated measurements were conducted, and the data were processed using the Malvern Zetasizer software (v4.20).

#### 2.4.3. Western Blot (WB) Analysis

WB analyses were performed as previously reported [28] to detect the expression of TSG101 and β-actin in ADMSCs and their derived EVs. For each sample, 30 μg of total protein was loaded onto SDS-PAGE gels and transferred to PVDF membranes. After blocking with 5% BSA, the membranes were incubated overnight at 4 °C with primary antibodies against TSG101 (BIOSS, bs-1365R, Beijing, China, 1:1000 dilution) and β-actin (Proteintech, Wuhan, China, #66009, 1:5000 dilution), followed by HRP-conjugated secondary antibodies. Protein bands were visualized using enhanced chemiluminescence (ECL) reagents and imaged with a chemiluminescence detection system (AI800, Cytiva, Shanghai, China).

### 2.5. Proteomic Analyses of the ADMSC-EVs of Qinling Giant Panda and hMSC-EVs

The resulting EVs were sequenced using proteomic sequencing at Novo Zhiyuan Biotechnology (Beijing, China) for the original data and subsequently analyzed using Gallus gallus Uniprot _2021.7.15.fasta (3430 go to sequences). The mass spectrometry proteomics data have been deposited to the ProteomeXchange Consortium via the PRIDE partner repository with the dataset identifier PXD059496.

To compare species-specific or conserved protein components, we conducted a proteomic analysis of Qinling giant panda ADMSC-EVs and human MSC-derived EVs (hMSC-EV). The hMSC-EV reference fasta dataset was obtained from the ExoCarta database (http://www.exocarta.org/ (accessed on 14 March 2024)), which includes EV proteomes derived from various human MSC sources, such as bone marrow, adipose tissue, and umbilical cord. Due to the lack of a dedicated dataset for human fetal ADMSC-derived EVs, we broadened the search to encompass multiple MSC sources to ensure a more comprehensive comparison.

### 2.6. Cell Proliferation Test

Passage 3 giant panda skin fibroblasts were incubated into 96-well plates at a density of 3 × 10^3^ cells per well and divided into control (CON) and ADMSC-EV groups, with each group containing six compound samples. The cells in the ADMSC-EV group were treated with 50 μg/mL of EVs per well, while the cells in the CON group were treated with an equal volume of PBS. The treatment lasted for 24, 48, 72, and 96 h. The cell proliferation was assessed using a Cell Counting Kit-8 (CCK-8) assay (Mishu, Xian, China, #MI00615A), and the absorbance was measured at 450 nm using a multi-mode microplate reader (Spark, TECAN, Seattle, WA, USA).

### 2.7. Cell Scratch Assay

Passage 3 giant panda skin fibroblasts were seeded into 6-well plates at a density of 2 × 10^5^ cells per well. Following a cell confluence of approximately 80%, they were scratched vertically using a sterile pipette tip. EVs (50 µg/mL) were added to the ADMSC-EV group, while the CON group received an equivalent volume of PBS. Images were captured under a microscope (Axio Observer3, ZEISS, Oberkochen, Germany) at 0, 12, 24, and 36 h. The scratch wound area at each time point was measured using ImageJ software (version 1.8.0). Migration rate = (Initial scratch wound area − Final scratch wound area)/Initial scratch wound area.

### 2.8. Establishment of Skin Wound Model in Mice

To establish the skin wound model, male BALB/c mice (6–8 weeks old, 25 ± 2 g) were anesthetized with intraperitoneal injection of Avertin (4 mg/kg body weight). The back region was shaved to remove hair, and the skin was disinfected. A sterile 1 cm diameter circular template was placed on the dorsal skin to ensure precise marking, and the skin within the template was gently marked using a sterile cotton swab soaked in iodine. The full-thickness skin, including epidermis and dermis, was then carefully excised using ophthalmic scissors along the marked boundary.

For treatment, the ADMSC-EV group received multiple subcutaneous injections of 100 µg of EVs around the wound site using a microinjector. The PBS group was injected with an equivalent volume of PBS, while the CON group did not receive any treatment. The wound area was covered with a sterile Vaseline gauze to prevent drying and contamination.

A total of 10 mice were used per group. Wound healing was monitored and photographed on days 7, 14, and 21. ImageJ software was used to analyze the wound area and perform wound superposition analysis by manually outlining the wound margins. Wound healing rate = (Initial area − Current area)/Initial area, where the initial area refers to the wound area measured on day 0, and the current area refers to the wound area at each subsequent time point. To assess wound healing at different time points, mice were sacrificed in a staged manner: 3 mice per group on day 7, 3 mice per group on day 14, and the remaining 4 mice per group on day 21. Serum and skin samples were collected for subsequent analysis.

### 2.9. Histological Staining

The skin tissues of mice in each group were fixed in 4% paraformaldehyde for 24 h, paraffin-embedded, and sectioned. H&E staining (Solarbio, Beijing, China, #G1120) and Masson’s trichrome staining (Solarbio, Beijing, China, #G1340) were performed according to the manufacturer’s instructions.

To quantify the relative thickness of the stratum spinosum, high-resolution H&E-stained images of the epidermis were captured. Image analysis was performed using ImageJ software, and the thickness of the stratum spinosum was measured by drawing perpendicular lines from the basal layer to the granulosum layer. The relative thickness was calculated by dividing the stratum spinosum thickness by the total epidermal thickness. Five measurements were taken from different regions of each sample to ensure accuracy, and the average value was used for analysis.

The collagen-positive areas were identified by the characteristic blue coloration of collagen fibers in Masson’s trichrome stain, and the analysis was performed using ImageJ software. The relative collagen volume fraction (CVF) was calculated as the ratio of the collagen-positive area to the total tissue area. To ensure accuracy, five random regions were selected for each sample, and the average CVF value from these regions was used for further analysis.

### 2.10. Immunohistochemistry (IHC) Procedure

Paraffin-embedded tissue sections were deparaffinized in xylene and rehydrated through a graded ethanol series. Antigen retrieval was performed by immersing the sections in citrate buffer (pH 6.0) and heating in a microwave oven at 95 °C for 10 min, followed by cooling to room temperature. Endogenous peroxidase activity was blocked by incubating the sections in 3% hydrogen peroxide for 10 min at room temperature. After washing with PBS, non-specific binding was blocked by incubating the sections with 5% bovine serum albumin (BSA) for 30 min at room temperature. Primary antibodies were applied to the tissue sections overnight at 4 °C: PCNA (Proteintech, Wuhan, China, #10205-2-AP, 1:500 dilution) and CD31 (Proteintech, Wuhan, China, #28083-1-AP, 1:500 dilution). Following PBS washes, the sections were incubated with an HRP-conjugated secondary antibody for 30 min at room temperature. DAB (3,3′-diaminobenzidine) was used as the chromogen for signal detection, and the nuclei were counterstained with hematoxylin. Finally, the sections were dehydrated, mounted, and examined under a light microscope (Ni-U, Nikon, Tokyo, Japan).

### 2.11. Enzyme-Linked Immunosorbent Assay (ELISA)

To assess the impact of ADMSC-EVs on inflammation in mice with skin defects, the concentrations of TNF-α and IL-6 in the serum of mice were measured using ELISA kits (KE10007 and KE10002, Proteintech, Wuhan, China) according to the manufacturer’s instructions.

### 2.12. Statistical Analysis

Data are expressed as mean ± standard error of the mean (SEM), and all data represent at least three technical replicates. GraphPad Prism (version 8.0) was used to analyze the data. One-way analysis of variance (ANOVA) was used to analyze statistical differences: * *p* < 0.05, ** *p* < 0.01, *** *p* < 0.001, and “ns” indicates no significant difference.

## 3. Results

### 3.1. EV Isolation and Characterization

**Figure 1:**
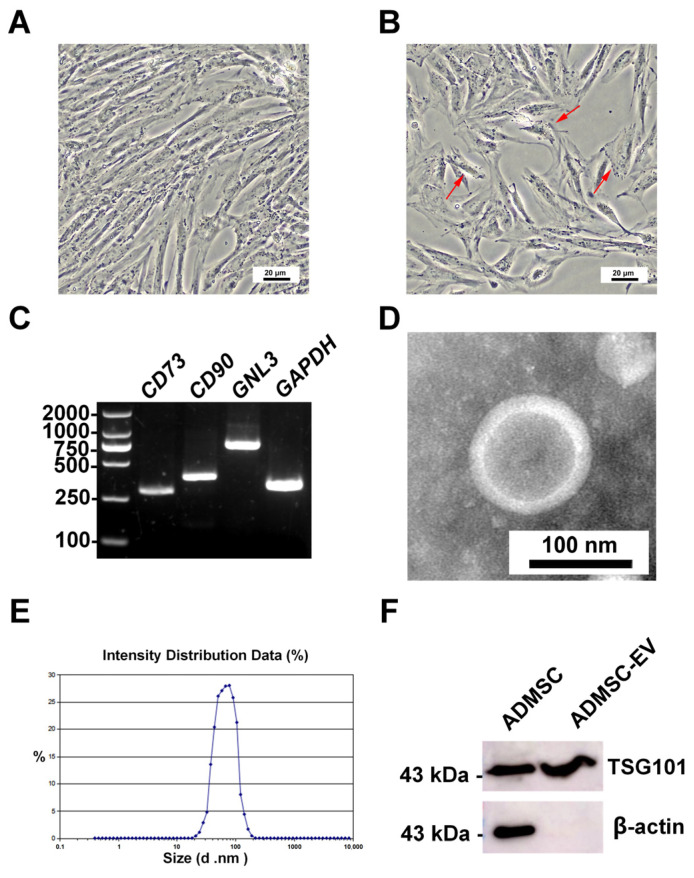
Morphology of the ADMSCs and characterization of EVs. (**A**) Passage 3 cells, cultured for 24 h; (**B**) Passage 8 cells, cultured for 24 h (red arrows indicate irregularly shaped ADMSCs); (**C**) RT-PCR analysis of *CD73*, *CD90*, and *GNL3*; (**D**) electron microscopy images of the ADMSC-EVs (2% uranium staining); (**E**) EV particle size distribution by Zetasizer; (**F**) WB results of the TSG101 and β-actin protein in giant panda ADMSCs and their EVs.

The giant panda ADMSCs exhibited uniform growth after 24 h of adherent culture, initially displaying an elongated fusiform shape (Figure 1A). However, after multiple passages, the cells in passage 8 began to adopt an irregular morphology (Figure 1B). RT-PCR analysis revealed that the giant panda ADMSCs expressed typical mesenchymal stem cell markers, including *CD73*, *CD90*, and *GNL3*. The PCR products migrated to the expected positions on the gel, confirming the accuracy and specificity of the amplification. (Figure 1C). The transmission electron microscopy showed that the obtained EVs displayed spherical structures with smooth edges and a bilayer membrane configuration (Figure 1D). The Zetasizer particle size analysis indicated that the EVs primarily had diameters ranging from 30 to 200 nm (Figure 1E). Western blot analysis confirmed that both the giant panda ADMSCs and their EVs expressed the specific surface marker TSG101. In contrast, only the cell group expressed the cytoskeletal protein β-actin, while no β-actin band was detected in the EV group (Figure 1F).

### 3.2. Proteomic Analysis of the ADMSC-EVs

**Figure 2:**
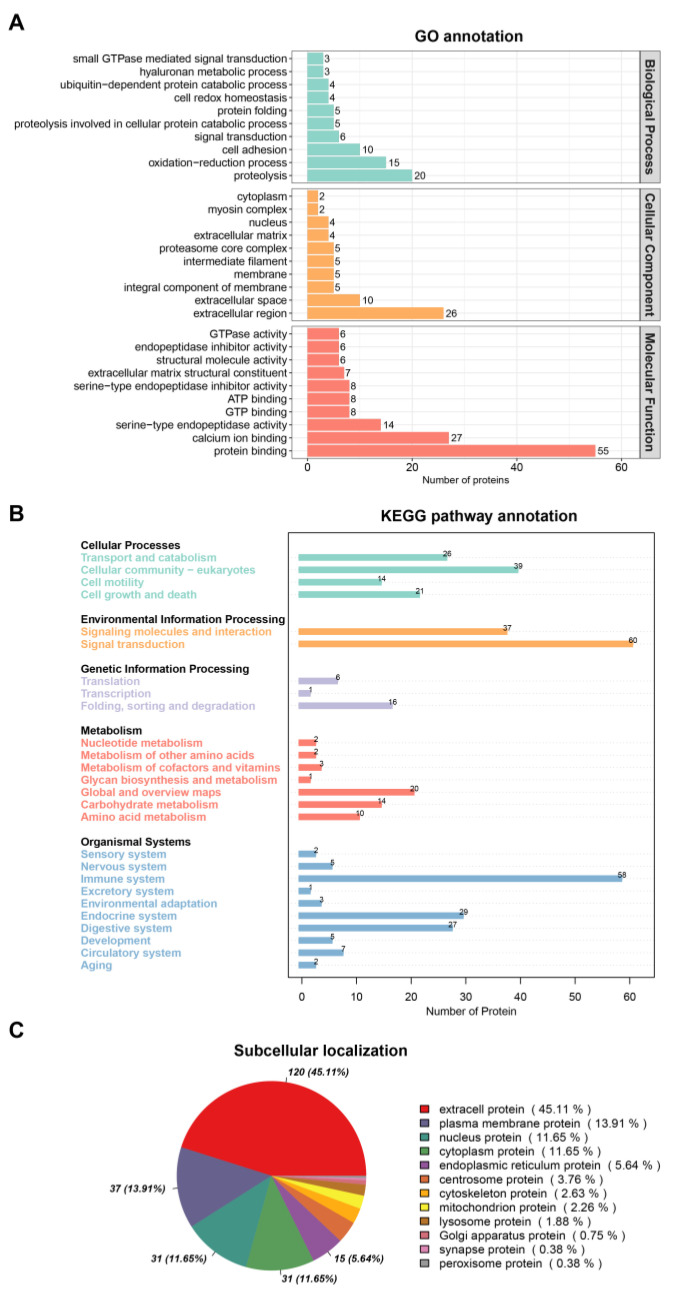
Proteomic analyses of the ADMSC-EVs. (**A**) GO analysis; (**B**) KEGG analysis; (**C**) subcellular localization analysis.

The proteomic analysis detected 301 proteins in the ADMSC-EVs of the Qinling giant panda. The Gene Ontology (GO) analysis revealed that these proteins were primarily involved in biological processes such as redox and cell adhesion. The cell classification proteins are located in the membrane and extracellular matrix, and their molecular functions primarily involve ATP binding and calcium ion binding (Figure 2A). The Kyoto Encyclopedia of Genes and Genomes (KEGG) analysis showed that signaling pathways, such as transport and catabolism, signal transduction, immune system, and amino acid metabolism, were concentrated (Figure 2B). The subcellular localization analysis of the detected proteins showed that they were localized in the extracellular and plasma membranes, followed by the nucleus and cytoplasm (Figure 2C).

### 3.3. Proteomic Comparison Between ADMSC-EVs and hMSC-EVs

**Figure 3:**
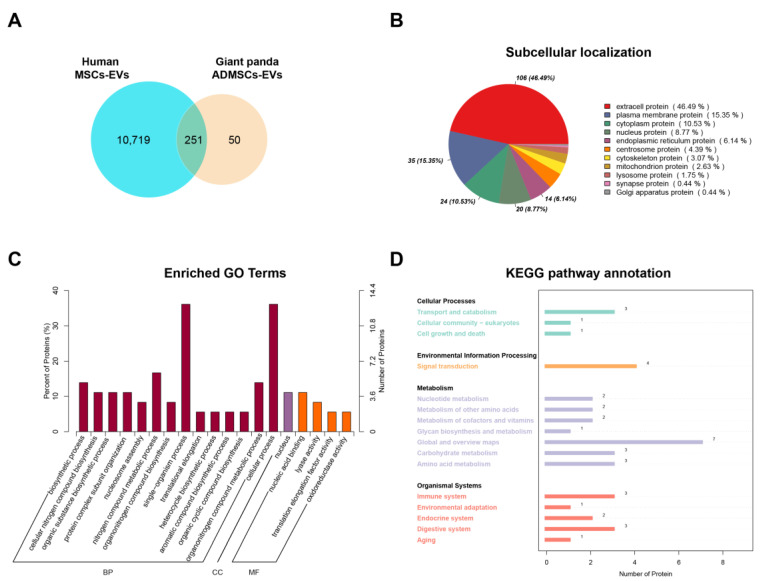
Comparative proteomic analyses of the human MSC-EVs and Qinling giant panda ADMSC-EVs. (**A**) Venn diagram of protein numbers in two species of EV; (**B**) subcellular localization of shared proteins; (**C**) GO analysis of the unique proteins in the giant panda ADMSC-EVs; (**D**) KEGG analysis of the unique proteins in the giant panda ADMSC-EVs.

The hMSC-EV database was obtained from ExoCarta, and we analyzed the differences between Qinling giant panda ADMSC-EVs and hMSC-EVs using a Venn diagram. A total of 10,970 proteins were identified in hMSC-EVs, with 301 proteins found in the ADMSC-EVs. Among them, 251 proteins were shared, and 50 proteins were unique to ADMSC-EVs (Figure 3A). The subcellular localization analysis of the shared proteins revealed that they were primarily localized in the extracellular and plasma membranes, followed by the cytoplasm and nucleus (Figure 3B). The unique proteins of ADMSC-EVs primarily participated in molecular functions and biological processes, including amino acid metabolism, nucleic acid binding, and biosynthesis (Figure 3C). Moreover, the KEGG analysis results indicated that they were concentrated in signaling pathways such as transport and catabolism, signal transduction, and the immune system (Figure 3D).

### 3.4. Morphological Observations of the Primary Skin Fibroblasts of Qinling Giant Panda

**Figure 4:**
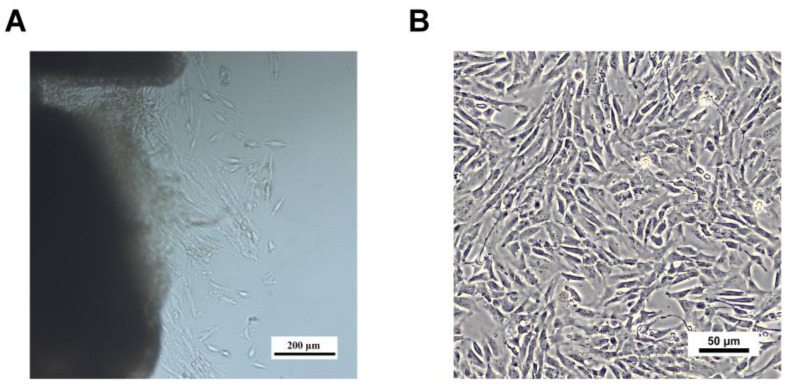
Morphological characteristics of the skin fibroblasts of Qinling giant panda primary cells. (**A**) Tissue block culture on day 6. (**B**) Passage 2 skin fibroblasts, cultured for 24 h.

The skin fibroblasts from Qinling giant pandas were isolated using tissue block culture. On day 6, the cells had gradually migrated out, exhibiting an irregular, flat morphology, with some cells beginning to elongate into a spindle shape (Figure 4A) When cultured to the second passage, most of the skin fibroblasts exhibited a spindle-shaped morphology with clear boundaries and were aligned in parallel (Figure 4B).

### 3.5. Effects of ADMSC-EVs on the Proliferation and Migration of Qinling Giant Panda Skin Fibroblasts

**Figure 5:**
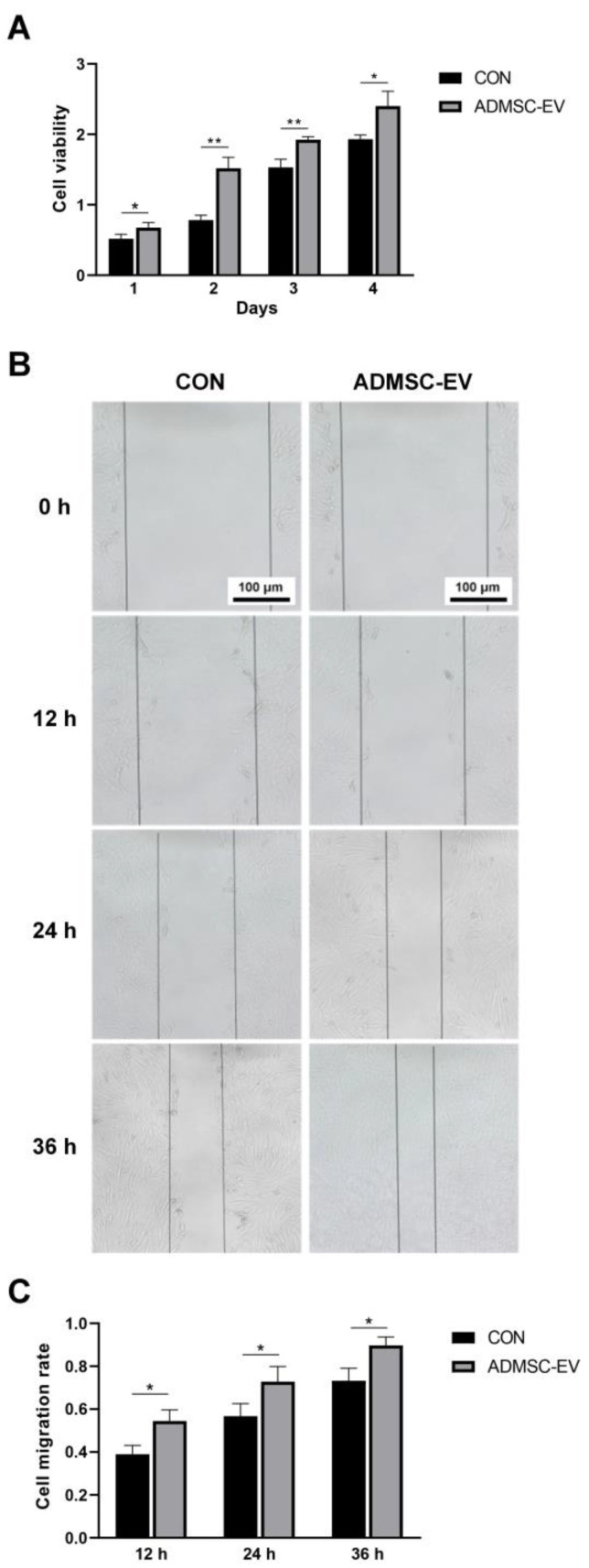
Proliferation and migration of skin fibroblasts treated with ADMSC-EVs. (**A**) Proliferation of skin fibroblasts following EV treatment (* *p* < 0.05, ** *p* < 0.01); (**B**) skin fibroblast migration following ADMSC-EV treatment; (**C**) cell migration rate statistics (* *p* < 0.05).

The proliferation rate of Qinling giant panda skin fibroblasts treated with ADMSC-EVs was significantly higher than that of the CON group, indicating that the ADMSC-EVs promoted the proliferation of skin fibroblasts (Figure 5A). The effects of the ADMSC-EVs on the migration ability of Qinling giant panda skin fibroblasts were determined using a cell scratch assay. At 0 h, the cell scratch widths in the CON and ADMSC-EV groups were similar. However, the migration rate of the ADMSC-EV group was significantly higher than that of the CON group at 12, 24, and 36 h (Figure 5B,C).

### 3.6. ADMSC-EVs Promote the Repair of Skin Injury in Mice

**Figure 6:**
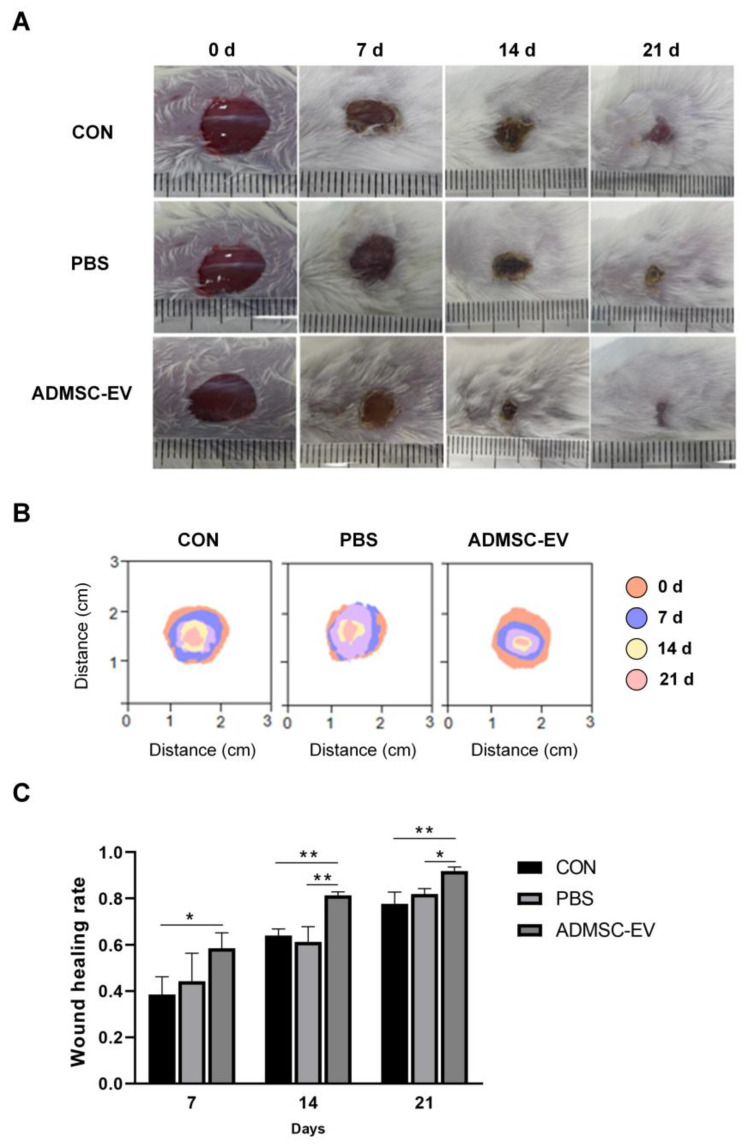
Results of skin healing in mice. (**A**) Records of skin injury in mice; (**B**) wound superposition in mice at different stages; (**C**) comparison of the wound healing rates of mice in each group (* *p* < 0.05, ** *p* < 0.01).

After 7 days of healing, all groups showed scab formation, with thin scabs, slight redness, and swelling surrounding the wounds (Figure 6A,B). The ADMSC-EV group exhibited a significantly higher wound healing rate compared to the CON group, although no statistical difference was observed between the ADMSC-EV and PBS groups (Figure 6C). After 14 days of healing, the wounds in the ADMSC-EV group showed a greater degree of epithelialization and a higher healing rate compared to both the CON and PBS groups (Figure 6A,C). Similarly, by day 21, the wounds in the ADMSC-EV group were almost completely healed, with significantly smaller wound sizes and higher healing rates compared to both the CON and PBS groups (Figure 6A–C).

### 3.7. ADMSC-EVs Promote the Repair of Skin Damage in Mice in Various Ways

**Figure 7:**
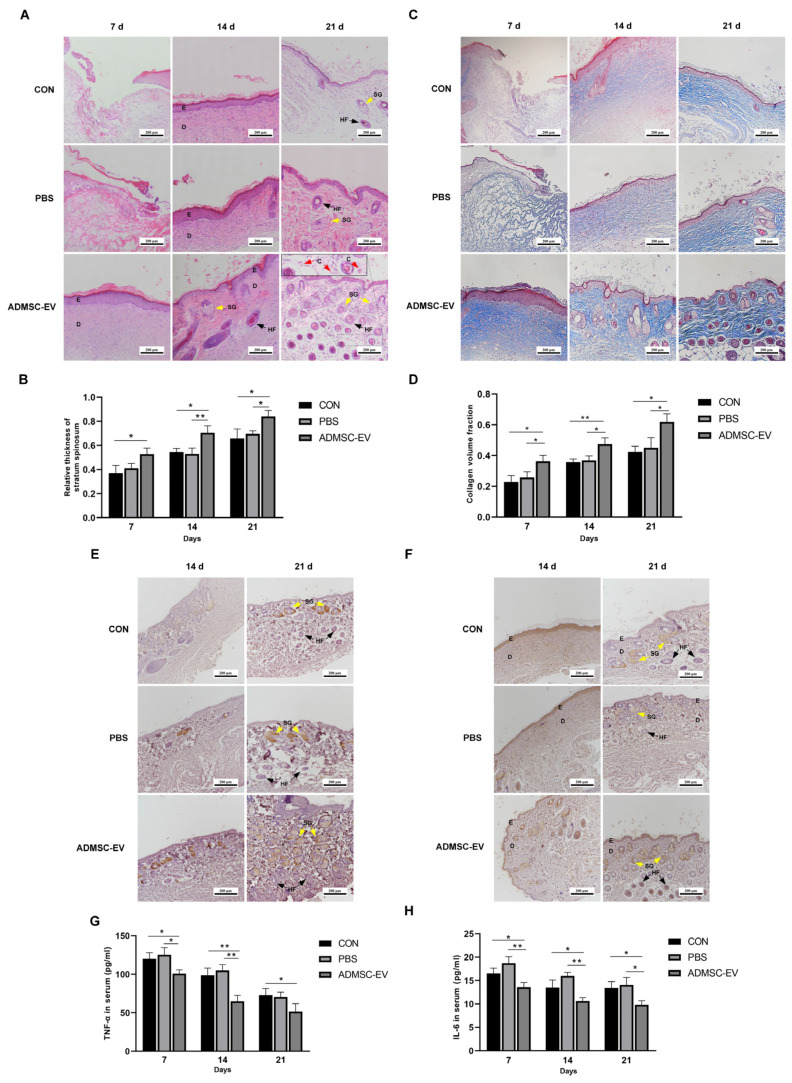
ADMSC-EVs promote the repair of skin damage in mice through multiple pathways. (**A**) Comparison of H&E staining results on the wounds of the three groups of mice; (**B**) comparison of the relative acanthoderm thickness of the wounds of the three groups of mice (* *p* < 0.05, ** *p* < 0.01); (**C**) comparison of collagen arrangements in the three groups of mice using Masson’s trichrome staining; (**D**) comparison of collagen volume fraction in the three groups of mice at different times (* *p* < 0.05, ** *p* < 0.01); (**E**) PCNA expression in the wounds of mice in the three groups; (**F**) CD31 expression in the three mice groups; (**G**) comparison of serum TNF-α content in the mice (* *p* < 0.05, ** *p* < 0.01); (**H**) comparison of serum IL-6 content in the mice (* *p* < 0.05, ** *p* < 0.01); E: epidermis; D: dermis; SG (yellow arrows): sebaceous glands; HF (black arrows): hair follicles; C (Red arrows): capillaries (n = 3–4).

The H&E staining revealed that on day 7, the ADMSC-EV group exhibited a well-defined epidermal–dermal junction with densely arranged cells, indicating an enhanced early-stage tissue organization. At this stage, the relative thickness of the stratum spinosum in the ADMSC-EV group was higher compared to the CON group. By day 14, while the CON and PBS groups began to show a clear separation between the epidermis and dermis, only the ADMSC-EV group presented newly formed hair follicles and emerging sebaceous glands. On day 21, the ADMSC-EV group displayed a markedly higher number of visible hair follicles and sebaceous glands compared to the other groups, along with increased capillary formation within the dermis, suggesting that ADMSC-EV treatment significantly promotes skin appendage regeneration and angiogenesis (Figure 7A). Additionally, the relative thickness of the stratum spinosum in the ADMSC-EV group remained elevated compared to both the CON and PBS groups, further supporting the positive impact of ADMSC-EVs on epidermal regeneration (Figure 7B). The Masson’s trichrome staining results revealed that the collagen fibers gradually increased with skin healing. Compared to the CON and PBS groups, the collagen fibers in the ADMSC-EV group were thicker and more tightly arranged (Figure 7C). The collagen volume fraction in the EV group was higher than that in the CON and PBS groups at each stage (Figure 7D).

PCNA and CD31 are marker proteins that reflect cell proliferation and angiogenesis during wound healing [29,30]. In all three groups, the expressions of PCNA and CD31 were observed after 14 and 21 days of healing. The PCNA expression was primarily localized in the sebaceous gland cells. For all three groups, the number of PCNA-positive cells significantly increased by day 21 of treatment compared to day 14, indicating a gradual increase in PCNA expression as the wound healed. Notably, the ADMSC-EV group exhibited higher levels of PCNA expression than the CON and PBS groups on both days 14 and 21, highlighting the potential advantage of ADMSC-EVs in promoting cell proliferation (Figure 7E). CD31 was extensively expressed in the epidermal and dermal layers of the mouse skin. By day 21, the number of CD31-positive cells in both the CON and PBS groups decreased compared to that at day 14, particularly within the epidermal layer. In the ADMSC-EV group, the expression of CD31 in both the dermal and epidermal layers remained consistently high. On day 21, this expression was significantly higher compared to the CON and PBS groups, particularly in the deeper hair follicles (Figure 7F).

As the healing process progressed, the concentrations of TNF-α and IL-6 in the serum of the three groups of mice gradually decreased. Notably, on days 7, 14, and 21, the levels of TNF-α and IL-6 in the serum of the ADMSC-EV group were significantly lower than those in the CON and PBS groups. Therefore, the ADMSC-EVs could effectively regulate the inflammatory response in the wound area of mice and reduce the infiltration and production of inflammatory mediators (Figure 7G,H).

## 4. Discussion

With its unique appearance and small population size, the Qinling giant panda has immense potential for conservation. However, its survival is threatened by numerous challenges, including trauma and infections from various causes [31,32,33]. hMSC-derived extracellular vesicles are beneficial for wound repair and tissue regeneration, and EV therapy has fewer side effects and is more effective than traditional wound treatment methods and cell therapy [34]. To date, no research has explored the effect of extracellular vesicle therapy on wound repair in giant pandas owing to their unique physiological characteristics and endangered status. Therefore, this study isolated giant panda ADMSC-EVs, conducted a proteomic analysis to compare them with hMSC-EVs, and investigated their effects on the proliferation and migration ability of skin fibroblasts. A mouse model of skin trauma was established to examine the in vivo effects of the ADMSC-EVs on wound healing.

Currently, EV-based treatments for skin injuries are applied in regenerative medicine. EV separation is vital for these treatments, and the primary techniques for EV separation include density gradient centrifugation, ultrafiltration, precipitation, immunoadsorption, size exclusion, and ExoQuick extraction. In particular, high-speed centrifugation methods are commonly used for this purpose [35,36]. Herein, the morphology and diameter of ADMSC-EVs from the Qinling giant panda were consistent with the classical morphological description of EVs [37]. Moreover, the Western blot results revealed that they expressed TSG101, indicating that the ADMSC-EVs from the Qinling giant panda were successfully isolated.

The subcellular localization results of the ADMSC-EVs indicated that most of the proteins were located in the nucleus and cytoplasm. The proteins shared by both ADMSC-EVs and hMSC-EVs were concentrated in the cell plasma membrane and extracellular matrix and were involved in cell adhesion, redox, and other biological processes, as well as ATP binding, calcium ion binding, and other molecular functions. The unique proteins of ADMSC-EVs were involved in molecular functions and biological processes, including amino acid metabolism, nucleic acid binding, and biosynthesis, and were concentrated in signaling pathways, such as transport and catabolism, signal transduction, and the immune system. These results confirmed that giant panda ADMSC-EVs and hMSC-EVs have common characteristics and similar functions, which is consistent with existing research views [38,39]. Previous research has demonstrated that ADMSC-EVs can promote coagulation and cell adhesion [40], thus promoting skin healing and confirming the feasibility of this approach in giant pandas.

The internalization of ADMSC-EVs by fibroblasts can stimulate the cell cycle of fibroblasts, thus promoting cellular collagen synthesis, migration, and proliferation [41]. In addition, ADMSC-EVs stimulate the expression of genes associated with fibroblast proliferation [42]. Herein, the ADMSC-EVs in the giant panda skin fibroblast culture medium significantly promoted the proliferation and migration of skin fibroblasts, demonstrating the effectiveness of ADMSC-EV therapy in promoting wound healing in giant pandas at the cellular level.

Studies using both autologous and allogeneic MSCs have shown promising results; however, when the conditioned media of MSCs are used in chronic wounds, the effect and regenerative capacity are similar or higher than those of MSCs [43]. In this study, a mouse skin injury model was used to evaluate the effects of ADMSC-EVs on wound healing in giant pandas. The results showed that ADMSC-EVs promoted faster wound healing; improved epidermal repair, collagen deposition, and hair follicle formation; and enhanced blood vessel formation. The IHC results showed that the expression levels of PCNA and CD31 in the EV group were higher than those in the CON and PBS groups. The enhanced vascularization likely contributed to improved tissue regeneration by ensuring better oxygen and nutrient supply [44]. Thus, the ADMSC-EVs promoted wound cell proliferation and angiogenesis, confirming that ADMSC-EVs from giant pandas can promote wound recovery in vivo.

Studies have shown that particular substances within MSC-EVs, such as miR-132, can increase the expression of IL-10 and reduce the levels of NF-κB, IL-6, and IL-1β, which is conducive to the regression of inflammation and promotion of wound healing [45,46]. In this study, ADMSC-EVs were injected into the wound, and the inflammatory cytokines TNF-α and IL-6 were significantly reduced in the EV group. Thus, the ADMSC-EVs effectively regulated the inflammatory response at the wound, shortened the inflammatory period to facilitate wound healing, and inhibited inflammatory responses in the process of promoting wound healing. These results confirm the potential clinical application of ADMSC-EVs.

Previous studies have shown that stem cells and their EVs derived from different donors can exhibit varying effects [47]. A limitation of this study is the variability between different donors, which could result in differing effects and complicate the interpretation and generalization of our results. Furthermore, the restricted availability of donor tissues may limit the broader applicability of the findings. While the current applications are primarily confined to animal models, further research is required to translate these results into practical applications for giant pandas. Future studies should focus on establishing a robust and safe application of ADMSC-EV therapy in giant pandas, while also considering donor variability and evaluating its potential for clinical use.

## 5. Conclusions

In summary, we successfully isolated and characterized ADMSC-EVs from Qinling giant pandas and conducted proteomic analyses to detect 301 proteins that were primarily involved in cell adhesion and transport, catabolism, immune system support, and other signaling pathways. Compared to the hMSC-EVs, 50 proteins unique to ADMSC-EVs were identified. They primarily participated in cell transport, catabolism, signal transduction, the immune system, and other signaling pathways. Subsequently, we demonstrated that the ADMSC-EVs enhanced cell viability and improved the migration and proliferation capacity of skin fibroblasts from Qinling giant pandas. The ADMSC-EVs of Qinling giant pandas promoted skin healing in mice, providing a theoretical basis for the clinical application of giant panda EVs and the protection of germplasm resources.

## Data Availability

The original contributions presented in this study are included in the article/Supplementary Materials. Further inquiries can be directed to the corresponding authors.

## Supplementary Materials

The following supporting information can be downloaded at: https://www.mdpi.com/article/10.3390/ani15091270/s1, Table S1: Primer sequences for RT-PCR.
